# The Evolution of Mechanical Properties and Cellular Structure of Apples During Freeze Drying Combined with Hot Air Drying (FD-HAD) Process

**DOI:** 10.3390/foods13233951

**Published:** 2024-12-07

**Authors:** Lili Li, Mengmeng Yang, Lewen Zhu, Wenchao Liu, Linlin Li, Weiwei Cao, Junliang Chen, Linlin Zhao, Chung Lim Law, Tongxiang Yang, Guangyue Ren, Xu Duan

**Affiliations:** 1 College of Food and Bioengineering, Henan University of Science and Technology, Luoyang 471000, China; li_lili0613@163.com (L.L.); 15839562251@163.com (M.Y.); zlw024527@163.com (L.Z.); wen_chaoliu@163.com (W.L.); linlinli2020@126.com (L.L.); caoweiwei@haust.edu.cn (W.C.); junliangchen@126.com (J.C.); txyamy@163.com (T.Y.); rgy@haust.edu.cn (G.R.); 2 Postdoctoral Practice Innovation Base, Luohe Vocational Technology College, Luohe 462002, China; 3 Henan Nanjiecun (Group) Co., Ltd., Luohe 462600, China; 4 College of Tourism and Culinary Science, Yangzhou University, Yangzhou 225127, China; zhaoll89@163.com; 5 Department of Chemical and Environmental Engineering, Malaysia Campus, University of Nottingham, Semenyih 43500, Selangor, Malaysia; chung-lim.law@nottingham.edu.my

**Keywords:** freeze drying, hot air drying, glass transition temperature, viscoelastic characteristics, pectin content, pectin esterification degree

## Abstract

Apples are one of the most popular fruits in the world and have a significant share in domestic and international fruit production. Drying is a common method used to extend the shelf life of apples. However, it also induces irregular morphological changes in apples, which are essential to maintaining the structural integrity of the material. Therefore, it is necessary to understand the effect of cellular changes at the microscopic level on the macroscopic deformation of the material during drying. In this paper, the evolution of cell wall pectin fractions and viscoelastic properties of apples during freeze drying combined with hot air drying was investigated. The findings indicated that during the HAD stage, a decrease in the relaxation modulus (*E*_1_) of the samples was observed in the compression tests when the sample temperature was significantly higher than the glass transition temperature (*T*_g_). As the difference between the two decreased, the samples exhibited increased stiffness and higher *E*_1_. The results of the pectin content analysis showed that the HAD process accelerated the loss and degradation of water-soluble pectin in the samples with high moisture content at the transition point. Simultaneously, the esterification degree of chelator-soluble pectin increased, leading to a reduction in the support provided to the cellular structure of the samples, which consequently affected their mechanical properties. These findings may provide valuable information for the application of freeze drying combined with hot air drying in the efficient processing of dried fruit and vegetable products.

## 1. Introduction

Fresh apples have a moisture content of up to 80–85% and are categorized as perishable foods [1,2]. Drying is a common processing method employed to extend the shelf life of apples, thereby enhancing the convenience of storage and transportation [3]. Traditional drying methods, such as freeze drying (FD) and hot air drying (HAD), are commonly used to produce dried food products. FD is known as a drying technique that effectively preserves the original nutritional value and shape of fruits and vegetables [4]. However, a significant challenge associated with FD is its prolonged drying time, which leads to high energy consumption and increases operational costs [5,6]. HAD, on the other hand, is one of the most widely applied techniques in fruit and vegetable processing. Nevertheless, the browning and severe shrinkage that occur during the HAD process significantly deteriorate the appearance quality of the products, thereby limiting their economic value [7]. Studies had shown that the combination of FD with hot air drying (HAD) could reduce the energy consumption of the FD process while improving the quality of the final product [8]. The sample is freeze-dried for a certain period of time and then dried with hot air. This form of dehydration is called combined drying, and the time node at which the drying transition takes place is referred to as the transition point. However, the large-scale application of freeze drying combined with hot air drying (FD-HAD) remains limited due to challenges in maintaining the appearance quality of the products [9].

In order to reduce the energy consumption of freeze-dried apples and simultaneously obtain dehydrated products with an optimal visual appearance, the research team recently employed FD-HAD for the dehydration of apples and investigated the shrinkage mechanism of the products during this process from the perspective of water migration [9]. Noticeably, the state and distribution of moisture within materials, and its interaction with the microstructure, can alter the mechanical and chemical properties of food products [10]. The mechanical properties of the samples are crucial for maintaining their structural integrity [11] During the drying process, the presence of internal temperature and moisture gradients induces the development of microstructural stresses, which result in structural changes within the sample. Additionally, as moisture is removed from the tissue, pores that were initially filled with water subject to tension, causing the solid matrix to contract inward. Stronger mechanical properties in the sample can resist the external forces generated by heat and mass transfer, thereby reducing changes in the sample’s shape and size [11,12,13]. Variations in the mechanical properties of samples during drying will affect the extent of deformation the material undergoes. Zhang et al. [14] observed that at low moisture content, the material continuously transformed from a highly elastic state to a glassy state, accompanied by an increase in rigidity. This transformation counteracted some of the capillary forces and prevented further shrinkage. Understanding the evolution of mechanical properties during drying is essential for elucidating the underlying mechanisms of sample shrinkage. Such insights can contribute to the precise regulation of deformation during drying, ensuring better control over the final product’s structural and functional properties.

High-moisture fruit and vegetable materials are typically regarded as viscoelastic, exhibiting both elastic and viscous properties when subjected to mechanical stress. The viscoelasticity of these materials is contingent upon structural alterations at the cellular level, which are influenced by cell expansion pressure, cell-to-cell adhesion, and cell wall rigidity. In particular, cell expansion pressure is dependent on moisture, while intercellular adhesion and cell wall rigidity are determined by the polysaccharides present in the cell wall [15,16,17]. Studies conducted by Bai et al. [1] had confirmed that cell wall properties significantly affect the porosity and shrinkage of dried products. The cell walls of plant cells consist of a complex matrix of polysaccharides, including lignin, cellulose, hemicellulose, and pectin. Pectin serves as a cross-linking agent within the cell wall, contributing to the overall viscoelasticity. Additionally, pectin can influence mechanical properties through direct interaction with other cell wall polysaccharides or by regulating cell wall porosity and moisture content [18]. Since the mechanical strength of the sample is linked to the final appearance of the products, the alterations in pectin structure may thus impact the preservation of sample morphology during drying, which in turn affects the quality of the dried product and consumer satisfaction [13]. In contrast, cellulose and lignin, which serve as the primary structural components of the cell wall, are more stable during the drying process, while pectin is more sensitive to drying due to its complex structure. Consequently, an investigation into the changing pattern of pectin molecules during a combined drying process may provide insights into the reasons behind the alteration of viscoelasticity in the samples during drying.

Currently, studies on drying agricultural products mainly focus on the physicochemical properties of the final products, with limited attempts to understand the relationship between cellular changes at the microscopic level and macroscopic mechanical changes during the drying process. This study aimed to investigate the mechanical properties and pectin composition changes of apple during the HAD stage of the FD-HAD process and analyze the relationship between microstructural changes and macroscopic mechanical properties. These findings can provide a reference for understanding the mechanism of volumetric shrinkage in the combined drying process.

## 2. Materials and Methods

### 2.1. Materials

Red Fuji apples purchased from Dazhang Supermarket in Luoyang City, Henan Province, were used in this study. Fresh apples of uniform size and maturity, free from pests and mechanical damage, were selected as raw materials, the initial dry basis moisture content of which was measured to be 6.45 ± 0.87 g/g. They were washed, peeled, and cut into cubes measuring 10 mm × 10 mm × 10 mm using a mold. The diced apple samples were soaked in a solution of 0.5% citric acid and 0.5% ascorbic acid for 20 min at room temperature to prevent browning phenomen. Afterward, the surface moisture was removed from the diced apples using filter paper, and they were subsequently subjected to a pre-freezing period of over 10 h at −25 °C in the freezer compartment of a refrigerator.

### 2.2. Drying Experiments

For the experiment, a freeze dryer (GIPP-4000, Shanghai Jipu Electronic Technology Co., Ltd., Shanghai, China) and a hot air dryer (DHG-9010, Shanghai Yiheng Technology Instrument Co., Ltd., Shanghai, China) were used. FD-HAD involves a two-stage drying process, and the drying procedure was referred to the method described by Zhang et al. [8] with appropriate modifications. In the FD stage, the apple cubes were subjected to vacuum freeze drying for durations of 11, 12, 13, and 14 h, respectively. The pressure of the drying chamber was set to 50 Pa, and the temperature of the cold trap was set to −70 °C. During the FD process, the temperature of the partition was set at 20 °C, while the desorption drying stage was conducted at 50 °C. The dry basis moisture content of the samples at the completion of FD was measured by the gravimetric method to be 1.00 ± 0.03, 0.76 ± 0.04, 0.53 ± 0.04, and 0.33 ± 0.03 g/g, respectively. Subsequently, the freeze-dried samples were subjected to HAD. The hot air temperature was set to 50 °C, and the samples were removed at 30 min intervals to determine the weight variations. The drying process was terminated when the moisture content was less than 0.08 g/g on a dry basis. The combined drying methods, involving transfer to hot air drying after freeze drying for 11, 12, 13, and 14 h, were designated as FD11-HAD, FD12-HAD, FD13-HAD, and FD14-HAD, respectively.

### 2.3. Determination of Glass Transition Temperature (T_g_)

The *T_g_* of samples with varying water contents during HAD were measured using a differential scanning calorimeter (DSC823e, Mettler-Toledo Instruments Co., Ltd., Shanghai, China) following the method described by Ren et al. [19]. The sample (10 mg) was placed in an aluminum crucible and sealed. The procedure was set to decrease the temperature at a cooling rate of 10 °C/min from 20 °C to −70 °C and then increase it to 70 °C at a rate of 10 °C/min after 2 min of stabilization. The *T_g_* of the apple could be observed from the heat flow diagram.

### 2.4. Determination of Viscoelastic Characteristics

The mechanical properties of the samples were determined in compression mode using a texture analyzer (TA-XT2i, Stable Micro Systems Ltd., Vienna Court, UK), following the methodology described by Li et al. [20]. For testing, the sample was compressed by a 2 mm diameter cylindrical probe at a speed of 1 mm/s and subjected to a constant strain of 15% for a duration of 2 min. The stress relaxation data obtained were fitted using a modified Maxwell model, allowing for the characterization of the stress relaxation properties of the samples.

$$\sigma =E_{0}\varepsilon_{0}+E_{1}\varepsilon_{0}e^{-t^{n}/\tau }$$

where *σ* represents the stress on the sample at time *t*, *E*_0_ and *E*_1_ are the residual modulus and relaxation modulus, respectively, *ε*_0_ is the strain compression. *τ* is the relaxation time, and *n* is the nonlinear index, taking values between 0 and 1.

### 2.5. Determination of Cell Wall Pectin

#### 2.5.1. Preparation of Alcohol Insoluble Residue (AIR)

The extraction of alcohol insoluble residues (AIR) from apples was conducted using the method described by Peng et al. [21]. Samples removed from the HAD stage were placed in a mortar and ground with the addition of liquid nitrogen. The powdered sample (1 g) was fully immersed in 30 mL of 95% ethanol and mixed for 10 min using a homogenizer (A25, Shanghai OUHOR Mechanical Equipment Co., Ltd., Shanghai, China). The resulting mixture was then filtered, and the residue was immersed again in 95% ethanol and remixed. After filtering, the residue was mixed in 20 mL of acetone for 10 min and subjected to a final filtration. The AIR extract was then dried at 40 °C until a constant weight was achieved. The dried AIR extract was subsequently ground uniformly and stored in a desiccator in anticipation of further analysis.

#### 2.5.2. Fractionation and Determination of Pectin

The pectin fractions were isolated and determined by referring to the method of Liu et al. [10] with slight modifications. The AIR (0.3 g) was dissolved in distilled water and extracted by shaking at 25 °C for 1 h. The supernatant was then separated by centrifugation to obtain water-soluble pectin (WSP). Subsequently, the residue was transferred to a solution of cyclohexane-trans-1,2-diamine tetraacetic acid (CDTA, 0.05 M, pH 6.5) containing 0.1 M potassium acetate and shaken for 6 h. After centrifugation for 15 min, the supernatant was separated to obtain chelator-soluble pectin (CSP). For Na_2_CO_3_-soluble pectin (NSP), the residue was diluted with a 0.05 M Na_2_CO_3_ solution (containing 0.02 M sodium borohydride) at 4 °C for 16 h, stirred at 25 °C for 6 h, and then centrifuged to obtain NSP. All pectin fractions were adjusted to a final volume of 50 mL at pH 6.5, and the corresponding galacturonic acid (GalA) content was determined using the sulfuric acid-carbazole method with a microplate photometer (Multiskan FC, Thermo Fisher Scientific Inc., Shanghai, China) at a wavelength of 530 nm.

#### 2.5.3. Determination of Pectin Esterification Degree

The degree of esterification of pectin was determined by reference to the method of Peng et al. [21] with slight modifications. The pectin fractions (1 mL) obtained from Section 2.5.2 were mixed with ethanol oxidase at a concentration of 1.0 U/mL, and the mixture was agitated at 25 °C for 15 min. Subsequently, 2 mL of glutaraldehyde solution was added and mixed at 58 °C for 15 min. The absorbance was measured at 412 nm (Multiskan FC, Thermo Fisher Scientific Inc., Shanghai, China) after the solution had cooled to room temperature.

### 2.6. Statistical Analysis

The data obtained from at least three replicate measurements were subjected to statistical analysis using one-way analysis of variance (ANOVA) in SPSS 26 software (SPSS, IBM Inc., Chicago, IL, USA). The results are presented as mean ± standard deviation, and Tukey’s post hoc test was employed to ascertain the significant differences between groups at a 95% confidence level (*p* < 0.05). Additionally, Origin 2023b software (Origin, OriginLab Corporation, Northampton, MA, USA) was utilized for data processing and visualization of the experimental results.

## 3. Results and Discussion

### 3.1. Glass Transition Temperature

Glass transition refers to the transformation of an amorphous material from a glassy to a rubbery state. The temperature at which this transition occurs is known as the glass transition temperature (*T_g_*) [22,23]. Significant changes in mechanical, thermal, and electrical properties occur when a food product converts between these states, making *T_g_* a critical factor in its processing and storage stability. As sample temperatures below *T_g_*, it remains in a glassy state, exhibiting rigidity and limited physicochemical changes. However, when the temperature of the sample exceeds *T_g_*, the sample transforms into an unstable rubbery state, accompanied by an exponential increase in molecular mobility and a significant decrease in viscosity. Such a process may result in the deterioration of the sample, which may manifest as structural collapse or shrinkage during processing or storage [7,24].

Table 1 shows the *T_g_* and actual temperature of central portion of the samples during the HAD stage of FD-HAD. As displayed in the table, the *T_g_* values of the samples exhibited a gradual increase as HAD progressed. A reduction in the moisture content of the samples could result in an increase in *T_g_*. In our previous study, we found that apple shrinkage occurred mainly in the HAD stage [9]. In the FD-HAD process, a high water content at the transition point indicated that the wet zone in the center of the sample at the initial stage of HAD contained more water, which implied that the *T_g_* of the sample was lower. This resulted in the sample being in a highly unstable rubbery state, exhibiting a greater degree of softness and proclivity for collapse and volumetric shrinkage. Therefore, the degree of volumetric shrinkage of the sample would be greater at this stage. When the temperature of the sample was below *T*_g_, the sample transitioned to a glassy state, exhibiting high stiffness and minimal volume shrinkage.

### 3.2. Viscoelasticity

**Figure 1:**
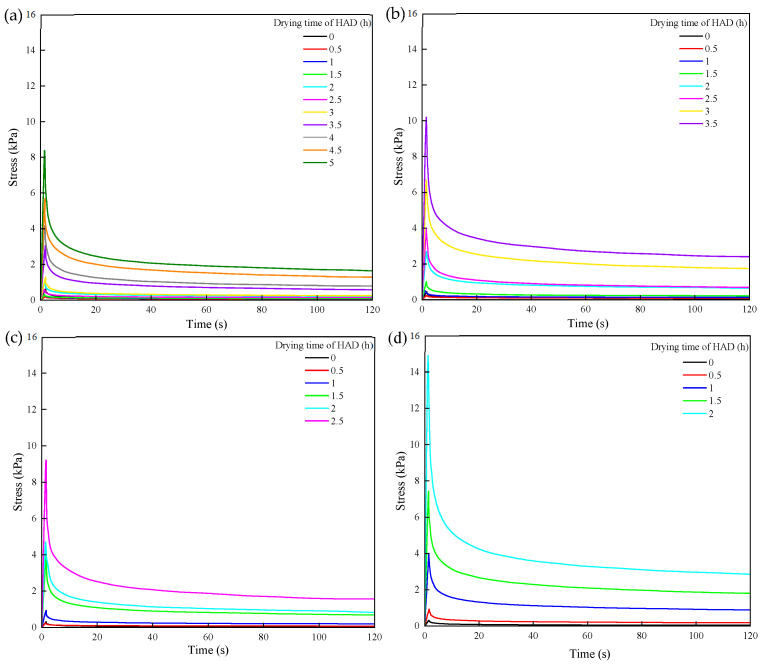
Stress relaxation curves of apple during the HAD stage under different FD-HAD drying schemes: (**a**) FD11-HAD, (**b**) FD12-HAD, (**c**) FD13-HAD, (**d**) FD14-HAD.

The stress relaxation behavior is closely related to the composition and structure of materials, reflecting their viscoelasticity [25]. Figure 1 illustrates the variations in stress relaxation behavior observed in the samples during the HAD stage under different FD-HAD schemes. In FD11-HAD and FD12-HAD, the stress relaxation curves of the samples in the initial stage of HAD exhibited a downward trend, accompanied by a reduction in relaxation stress. This phenomenon might be attributed to the higher moisture content present in the samples at the transition point, which exerted a pronounced plasticizing effect, thereby softening the samples [26]. As the drying time increased, the stress relaxation curves of the samples shifted upward, indicating an increase in sample hardness due to the continuous removal of moisture, which subsequently elevated the relaxation stress within the samples.

The Maxwell model is one of the simplest and most fundamental mathematical models for describing the mechanical properties of viscoelastic solid materials [27]. It effectively captures the time-dependent stress relaxation characteristics. The fitted parameters, including the residual modulus (*E*_0_), relaxation modulus (*E*_1_), relaxation time (*τ*), and nonlinear index (*n*), characterize the stress relaxation behavior of the sample. The coefficient of determination (*R*^2^) serves as an effective indicator of the accuracy of the fitting results. The fitted parameters (*E*_0_, *E*_1_, *τ*, and *n*), along with the coefficient of determination (*R*^2^) of the model, are presented in Table 2. As illustrated in the table, the relaxation modulus (*E*_1_) of the samples exhibited a notable decline followed by an upward trend when the freeze drying time was less than 13 h. This was because the residual ice crystals present in the samples at the end of FD would provide part of the mechanical strength for the samples [28]. However, when HAD had been performed, the ice crystals melted and the plasticizing effect of liquid water softened the sample [26]. Furthermore, since the actual temperature of the sample was higher than its glass transition temperature, they were in a rubbery state, making them less capable of resisting external forces. As the HAD process continued, the water content within the samples gradually evaporated, resulting in an increase in the *E*_1_ value over time. Noticeably, the *E*_1_ of the material subjected to HAD after 14 h of FD was consistently elevated and exhibited no decrease. This might be due to the removal of most ice crystals during the FD process, which allowed for the formation of a rigid pore structure within the sample [29], thus preventing significant structural changes during the HAD stage. Therefore, an increased *E*_1_ value of the sample was observed.

The relaxation time (*τ*) is a parameter that reflects the rate of energy release. In different drying schemes, the value of *τ* exhibited a gradual decline as the drying process progressed. This phenomenon was found to be associated with the resistance of the macromolecules within the material during compression [20]. The longer the relaxation time, the longer it took for the molecular chains inside the sample to move to an equilibrium position, indicating a more elastic material [20,30,31]. The relaxation index (*n*) is an effective indicator of the integrity of the internal network structure of the material and its resistance to external forces [30]. As shown in Table 2, the relaxation index demonstrated a similar tendency as *E*_1_, initially decreasing and subsequently exhibiting a gradual increase as the drying process continued. This behavior may be attributed to the melting of ice crystals in the samples during the initial stage of HAD, as well as the migration of moisture and the collapse of pores, which resulted in the disruption of the internal structure and a concomitant reduction in resistance to external forces. As the drying process continued, the evaporation of water from the samples allowed for the gradual reconnection of the internal pore network, enhancing the samples’ resistance to external forces and leading to an increase in the relaxation index.

### 3.3. Pectin Content

**Figure 2:**
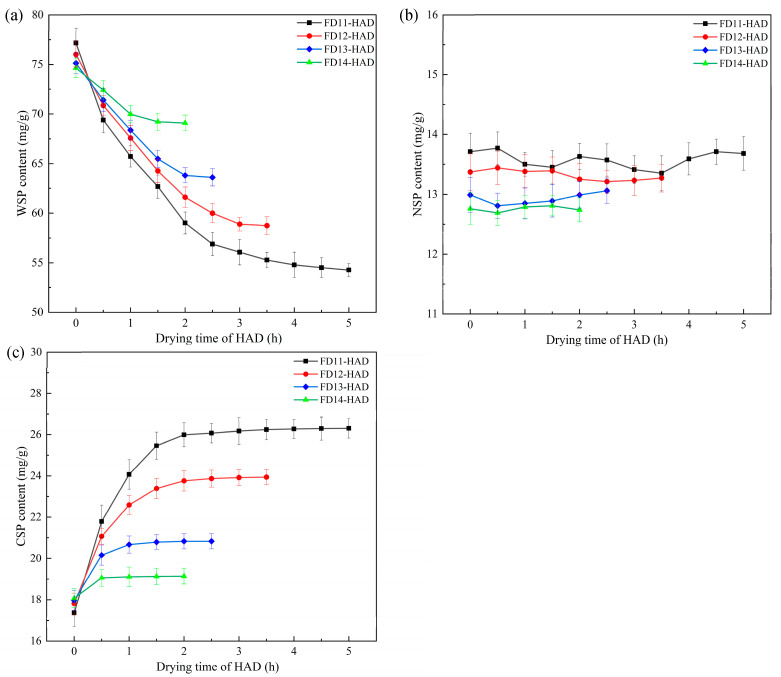
Changes of pectin content in apple during HAD stage under different FD-HAD drying schemes: (**a**) WSP, (**b**) NSP, (**c**) CSP.

Pectin is a diverse and complex biopolymer present in the cell wall which helps to enhance intercellular adhesion and cellular mechanical strength and prevents aggregation and collapse of the internal structure of the cell wall [10,32]. Figure 2 shows the alterations in the pectin content of the samples during the HAD stage under different FD-HAD drying schemes. The contents of three types of pectin (WSP, CSP, and NSP) in the samples were not significantly different at the transition point (*p* < 0.05). This was because the FD process caused less damage to the cellular structure of the samples, resulting in higher pectin retention [33]. Following the transition to the HAD stage, the content of WSP decreased while the CSP content demonstrated a gradual increase. In contrast, the content of NSP remained relatively stable during the HAD stage.

WSP is a pectin that relies on non-covalent and non-ionic bonds to loosely bind to the cell wall and participate in intercellular adhesion [34,35]. During the drying process, some of the WSP might be leached out with the apple juice, and the pectin fraction was degraded or transformed as the drying time increased, resulting in a lower WSP content [36]. Notably, higher moisture content at the transition point of combined drying corresponded to lower WSP content and higher CSP content in the samples at the end of the drying process. This phenomenon might be attributed to the prolonged heat drying, which increased water migration and led to accelerated loss and conversion of WSP [37]. Additionally, the decrease in WSP, along with the loss or depolymerization of pectin, resulted in a diminished supportive effect on the tissue [36]. This might be one of the factors contributing to the decrease in *E*_1_ of the samples during the initial stage of HAD.

### 3.4. Pectin Esterification Degree

**Figure 3:**
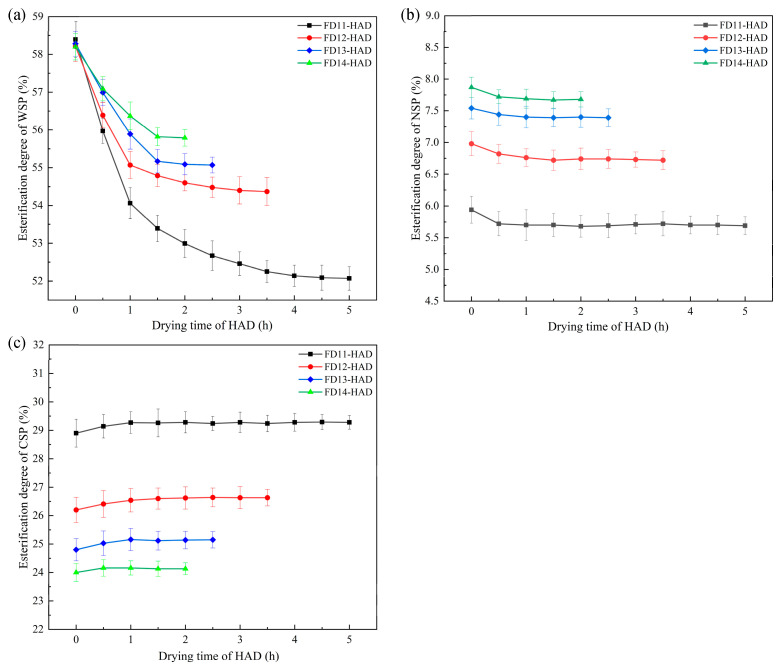
Changes in the esterification degree of pectin during the HAD stage of apple under different FD-HAD drying schemes: (**a**) WSP, (**b**) NSP, (**c**) CSP.

The degree of esterification is defined as the extent to which the polygalacturonic acid present in the primary chain of pectin is esterified [37]. The degree of esterification of pectin affects the hydrogen bonding between pectin molecules, which in turn affects the textural properties of fruits and vegetables [36]. Figure 3 illustrates the variation in pectin esterification degree of the samples during the HAD stage under different FD-HAD drying schemes. As shown in the figure, the degree of esterification of WSP in the samples exhibited a gradual decline, whereas the esterification degree of CSP and NSP remained relatively stable. This was attributed to the elevated activity of pectin methyl esterase at 50 °C, which exerted a considerable hydrolysis effect on the methyl ester group of WSP, thereby reducing the WSP esterification degree [37]. Furthermore, it was observed that the lower moisture content at the transition point corresponded to a lower degree of esterification of CSP. CSP is a pectin that interacts through ionic bonding, binding to the cell wall through calcium bridges and playing an important role in maintaining the cell wall integrity [38]. A lower degree of esterification in CSP resulted in more free carboxyl groups, facilitating cross-linking with metal ions in the tissues [21,39], which enhanced the tissue strength of the samples, resulting in a higher relaxation modulus.

## 4. Conclusions

Results showed that the central portion of the sample was in a rubbery state and exhibited increased malleability during the initial stage of HAD, while the relaxation modulus (*E*_1_) and relaxation index (*n*) of the samples exhibited a declining trend. As drying progressed, this region underwent a gradual transition to a glassy state, characterized by enhanced rigidity. The findings of the study on the alteration of pectin content and structure during the HAD stage indicated that the loss and degradation of WSP in the combined drying HAD stage, along with the elevated esterification degree of CSP, were pivotal factors contributing to the reduced *E*_1_ of the samples, which subsequently compromised their ability to support tissue strength. The generated knowledge might contribute to a deeper understanding of the mechanical and structural dynamics in combined drying processes. In future studies, we will focus on enhancing the crosslinking between Ca^2+^ and pectin molecules to increase sample tissue strength, providing strategies to mitigate shrinkage and deformation during the drying process.

## Figures and Tables

**Table 1:** The glass transition temperature changes in apple HAD stage with different FD-HAD drying methods.

Drying Time of HAD Stage (h)	Drying Schemes							
	FD11-HAD		FD12-HAD		FD13-HAD		FD14-HAD	
	*T_g_* (°C)	*T_w_* (°C)	*T_g_* (°C)	*T_w_* (°C)	*T_g_* (°C)	*T_w_* (°C)	*T_g_* (°C)	*T_w_* (°C)
0	−62.57 ± 2.21 ^i^	−13 ± 2 ^h^	−60.79 ± 2.67 ^f^	−8 ± 1 ^g^	−41.68 ± 2.10 ^e^	−3 ± 1 ^e^	−16.97 ± 1.29 ^d^	2 ± 1 ^d^
0.5	−59.98 ± 2.27 ^h^	28 ± 2 ^g^	−38.97 ± 2.19 ^e^	31 ± 2 ^f^	−12.87 ± 1.79 ^d^	30 ± 2 ^d^	33.1 ± 1.65 ^c^	40 ± 1 ^c^
1.0	−40.67 ± 2.17 ^g^	33 ± 1 ^f^	−10.11 ± 1.96 ^d^	35 ± 1 ^e^	21.98 ± 1.98 ^c^	35 ± 2 ^c^	48.39 ± 1.43 ^b^	44 ± 1 ^b^
1.5	−20.14 ± 1.28 ^f^	35 ± 1 ^e^	17.98 ± 1.08 ^c^	39 ± 1 ^d^	47.66 ± 1.87 ^b^	44 ± 1 ^b^	51.69 ± 1.65 ^a^	46 ± 1 ^a^
2.0	7.33 ± 1.01 ^e^	38 ± 1 ^d^	35.07 ± 1.41 ^b^	42 ± 1 ^c^	51.36 ± 1.70 ^a^	46 ± 1 ^a^	51.01 ± 1.57 ^a^	47 ± 1 ^a^
2.5	20.21 ± 1.01 ^d^	40 ± 1 ^c^	49.36 ± 1.89 ^a^	44 ± 1 ^b^	52.07 ± 1.60 ^a^	47 ± 1 ^a^		
3.0	34.98 ± 1.54 ^c^	43 ± 1 ^b^	51.97 ± 1.76 ^a^	46 ± 1 ^a^				
3.5	48.39 ± 1.65 ^b^	45 ± 1 ^ab^	52.68 ± 1.89 ^a^	47 ± 1 ^a^				
4.0	51.01 ± 1.39 ^a^	46 ± 1 ^a^						
4.5	52.07 ± 1.47 ^a^	47 ± 1 ^a^						
5.0	52.14 ± 1.69 ^a^	47 ± 1 ^a^						

Notes: *T_g_* and *T_w_* are the glass transition temperature and actual temperature of the sample center, respectively. Different letters indicate significant differences between different drying methods at different times (*p* < 0.05).

**Table 2:** Maxwell model parameters of stress relaxation of apple during the HAD stage under different FD-HAD drying schemes.

Drying Schemes	Drying Time of HAD Stage (h)	*E*_0_ (Mpa)	*E*_1_ (Mpa)	*n*	*τ* (s)	*R* ^2^
FD11-HAD	0	0.102 ± 0.04 ^i^	4.320 ± 1.32 ^f^	0.396 ± 0.02 ^a^	1.744 ± 0.23 ^a^	0.998
	0.5	0.355 ± 0.06 ^h^	0.964 ± 0.23 ^j^	0.281 ± 0.02 ^b^	1.634 ± 0.27 ^b^	0.993
	1.0	0.394 ± 0.11 ^h^	0.929 ± 0.32 ^j^	0.289 ± 0.03 ^c^	1.613 ± 0.23 ^c^	0.993
	1.5	0.745 ± 0.14 ^g^	1.811 ± 0.39 ^i^	0.288 ± 0.02 ^c^	1.456 ± 0.17 ^d^	0.991
	2.0	0.768 ± 0.22 ^g^	2.174 ± 0.65 ^h^	0.309 ± 0.01 ^d^	1.429 ± 0.19 ^e^	0.992
	2.5	0.923 ± 0.21 ^f^	3.111 ± 0.98 ^g^	0.322 ± 0.02 ^e^	1.428 ± 0.11 ^e^	0.991
	3.0	1.559 ± 0.43 ^e^	6.889 ± 2.11 ^e^	0.323 ± 0.01 ^f^	1.381 ± 0.12 ^f^	0.992
	3.5	3.572 ± 0.98 ^d^	18.464 ± 4.22 ^d^	0.330 ± 0.02 ^g^	1.332 ± 0.09 ^g^	0.992
	4.0	4.606 ± 1.22 ^c^	25.802 ± 5.87 ^c^	0.342 ± 0.02 ^h^	1.308 ± 0.09 ^h^	0.992
	4.5	7.261 ± 2.37 ^b^	35.222 ± 7.67 ^b^	0.345 ± 0.01 ^h^	1.308 ± 0.12 ^h^	0.996
	5.0	10.229 ± 2.98 ^a^	57.057 ± 12.25 ^a^	0.354 ± 0.02 ^i^	1.220 ± 0.07 ^i^	0.991
FD12-HAD	0	0.365 ± 0.09 ^g^	2.413 ± 0.88 ^g^	0.370 ± 0.02 ^a^	1.550 ± 0.21 ^a^	0.998
	0.5	0.450 ± 0.08 ^f^	1.289 ± 0.37 ^h^	0.289 ± 0.03 ^a^	1.500 ± 0.24 ^b^	0.991
	1.0	0.973 ± 0.10 ^e^	3.144 ± 0.99 ^f^	0.339 ± 0.03 ^b^	1.339 ± 0.29 ^c^	0.994
	1.5	1.471 ± 0.16 ^d^	6.369 ± 1.22 ^e^	0.350 ± 0.02 ^b^	1.297 ± 0.12 ^d^	0.993
	2.0	4.383 ± 0.67 ^c^	14.993 ± 2.21 ^d^	0.368 ± 0.01 ^c^	1.286 ± 0.11 ^e^	0.994
	2.5	4.392 ± 0.88 ^c^	23.851 ± 4.98 ^c^	0.379 ± 0.02 ^d^	1.266 ± 0.13 ^f^	0.990
	3.0	10.286 ± 2.37 ^b^	39.616 ± 6.34 ^b^	0.386 ± 0.01 ^e^	1.240 ± 0.09 ^g^	0.996
	3.5	14.295 ± 3.89 ^a^	60.989 ± 10.32 ^a^	0.391 ± 0.01 ^f^	1.212 ± 0.09 ^h^	0.990
FD13-HAD	0	0.021 ± 0.01 ^f^	2.223 ± 0.78 ^e^	0.336 ± 0.02 ^a^	1.627 ± 0.22 ^a^	0.992
	0.5	0.458 ± 0.05 ^e^	1.377 ± 0.39 ^f^	0.317 ± 0.03 ^b^	1.571 ± 0.21 ^b^	0.995
	1.0	1.226 ± 0.12 ^d^	5.355 ± 1.23 ^d^	0.346 ± 0.02 ^c^	1.323 ± 0.18 ^c^	0.997
	1.5	4.392 ± 0.37 ^c^	23.851 ± 3.28 ^c^	0.377 ± 0.01 ^d^	1.286 ± 0.18 ^d^	0.991
	2.0	5.436 ± 0.56 ^b^	30.261 ± 5.45 ^b^	0.390 ± 0.02 ^e^	1.286 ± 0.16 ^d^	0.994
	2.5	7.745 ± 1.26 ^a^	65.025 ± 11.11 ^a^	0.404 ± 0.01 ^f^	1.084 ± 0.08 ^e^	0.994
FD14-HAD	0	0.177 ± 0.02 ^e^	2.122 ± 0.11 ^e^	0.322 ± 0.03 ^a^	1.509 ± 0.19 ^a^	0.992
	0.5	1.447 ± 0.09 ^d^	6.085 ± 1.09 ^d^	0.336 ± 0.03 ^b^	1.503 ± 0.11 ^b^	0.995
	1.0	5.371 ± 1.21 ^c^	22.606 ± 3.37 ^c^	0.353 ± 0.01 ^c^	1.338 ± 0.09 ^c^	0.992
	1.5	9.358 ± 1.89 ^b^	44.881 ± 5.76 ^b^	0.391 ± 0.02 ^d^	1.234 ± 0.07 ^d^	0.994
	2.0	20.498 ± 2.76 ^a^	100.751 ± 20.28 ^a^	0.405 ± 0.01 ^e^	1.075 ± 0.07 ^e^	0.993

Note: Different letters represent significant differences between means (*p* < 0.05).

## Data Availability

The original contributions presented in the study are included in the article, further inquiries can be directed to the corresponding author.

## References

[1] Bai J.W., Zhang L., Aheto J.H., Cai J.R., Wang Y.C., Sun L., Tian X.Y. (2023). Effects of different pretreatment methods on drying kinetics, three-dimensional deformation, quality characteristics and microstructure of dried apple slices. Innov. Food Sci. Emerg. Technol..

[2] Joardder M.U.H., Brown R.J., Kumar C., Karim M.A. (2015). Effect of Cell Wall Properties on Porosity and Shrinkage of Dried Apple. Int. J. Food Prop..

[3] Kahraman O., Malvandi A., Vargas L., Feng H. (2021). Drying characteristics and quality attributes of apple slices dried by a non-thermal ultrasonic contact drying method. Ultrason. Sonochem..

[4] Chen B., Lin G., Amani M., Yan W. (2023). Microwave-assisted freeze drying of pineapple: Kinetic, product quality, and energy consumption. Case Stud. Therm. Eng..

[5] Huang L., Zhang M., Mujumdar A.S., Lim R.-X. (2011). Comparison of four drying methods for re-structured mixed potato with apple chips. J. Food Eng..

[6] Wu X.-F., Zhang M., Bhandari B., Li Z. (2019). Effects of microwave assisted pulse fluidized bed freeze-drying (MPFFD) on quality attributes of Cordyceps militaris. Food Biosci..

[7] Hou H.N., Chen Q.Q., Bi J.F., Wu X.Y., Jin X.W., Li X., Qiao Y.N., Lyu Y. (2020). Understanding appearance quality improvement of jujube slices during heat pump drying via water state and glass transition. J. Food Eng..

[8] Zhang L., Qiao Y., Wang C., Liao L., Liu L., Shi D., An K., Hu J., Xu Q. (2019). Effects of Freeze Vacuum Drying Combined with Hot Air Drying on the Sensory Quality, Active Components, Moisture Mobility, Odors, and Microstructure of Kiwifruits. J. Food Qual..

[9] Ren G., Zhu L., Duan X., Liu W., Li G., Wei X. (2024). Volume shrinkage mechanism for combined vacuum freeze drying-hot air drying of diced apples. Trans. Chin. Soc. Agric. Eng..

[10] Liu Y., Sun W., Li B., Wang Y., Lv W., Shang N., Li D., Wang L. (2022). Dehydration characteristics and evolution of physicochemical properties of Platycodon grandiflorum (Jacq. A.DC.) roots (PGR) during pulse-spouted microwave vacuum drying (PSMVD). Ind. Crops Prod..

[11] Joardder M.U.H., Kumar C., Karim M.A. (2018). Prediction of porosity of food materials during drying: Current challenges and directions. Crit. Rev. Food Sci. Nutr..

[12] Aprajeeta J., Gopirajah R., Anandharamakrishnan C. (2015). Shrinkage and porosity effects on heat and mass transfer during potato drying. J. Food Eng..

[13] Mahiuddin M., Khan M.I.H., Kumar C., Rahman M.M., Karim M.A. (2018). Shrinkage of Food Materials During Drying: Current Status and Challenges: Shrinkage of food materials during drying. Compr. Rev. Food Sci. Food Saf..

[14] Zhang L., Wang X., Wei Z., Sun C. (2016). Structural properties research of infrared radiation drying for carrot slices. Trans. Chin. Soc. Agric. Mach..

[15] Ortiz-Viedma J., Rodriguez A., Vega C., Osorio F., Defillipi B., Ferreira R., Saavedra J. (2018). Textural, flow and viscoelastic properties of Hass avocado (*Persea americana* Mill.) during ripening under refrigeration conditions. J. Food Eng..

[16] Lee J.W., Tan J., Waluyo S. (2016). Hysteresis characteristics and relationships with the viscoelastic parameters of apples. Eng. Agric. Environ. Food.

[17] Wu J., Guo K.Q. (2010). Dynamic viscoelastic behaviour and microstructural changes of Korla pear (Pyrus bretschneideri rehd) under varying turgor levels. Biosyst. Eng..

[18] Lahaye M., Falourd X., Laillet B., Le Gall S. (2020). Cellulose, pectin and water in cell walls determine apple flesh viscoelastic mechanical properties. Carbohydr. Polym..

[19] Ren G.Y., Zeng F.L., Duan X., Liu L.L., Duan B., Wang M.M., Liu Y.H., Zhu W.X. (2015). The Effect of Glass Transition Temperature on the Procedure of Microwave–Freeze Drying of Mushrooms (*Agaricus bisporus*). Dry. Technol..

[20] Li L., Ren X., Chen J., Cao W., Ren G., Bhandari B., Ren A., Duan X. (2022). Changes and relationships of viscoelastic and physical properties of Chinese yam during a novel multiphase microwave drying process. LWT-Food Sci. Technol..

[21] Peng J., Bi J., Yi J., Wu X., Zhou M., Zhao Y., Liu J.N. (2019). Characteristics of cell wall pectic polysaccharides affect textural properties of instant controlled pressure drop dried carrot chips derived from different tissue zone. Food Chem..

[22] Mahato S., Zhu Z., Sun D.-W. (2019). Glass transitions as affected by food compositions and by conventional and novel freezing technologies: A review. Trends Food Sci. Technol..

[23] Duan X., Ding L., Ren G., Liu L., Kong Q. (2013). The drying strategy of atmospheric freeze drying apple cubes based on glass transition. Food Bioprod. Process..

[24] Sablani S.S., Kasapis S., Rahman M.S. (2007). Evaluating water activity and glass transition concepts for food stability. J. Food Eng..

[25] Xu B., Li H., Zhang Y. (2013). An Experimental and Modeling Study of the Viscoelastic Behavior of Collagen Gel. J. Biomech. Eng..

[26] Ozturk O.K., Takhar P.S. (2020). Physical and viscoelastic properties of carrots during drying. J. Texture Stud..

[27] Zhou X., Yu D., Barrera O. (2023). Chapter Three—Mechanics constitutive models for viscoelastic solid materials: Development and a critical review. Adv. Appl. Mech..

[28] Sun Q., Zhang M., Mujumdar A.S., Yu D. (2022). Research on the vegetable shrinkage during drying and characterization and control based on LF-NMR. Food Bioprocess. Technol..

[29] Huang L., Lian M., Duan X., Li B., Yang S. (2021). Studies on the quality and moisture distribution of kiwifruit dried by freeze drying combined with microwave vacuum drying. J. Food Process Eng..

[30] Zhang H., Xiong Y., Bakry A.M., Xiong S., Yin T., Zhang B., Huang J., Liu Z., Huang Q. (2019). Effect of yeast β-glucan on gel properties, spatial structure and sensory characteristics of silver carp surimi. Food Hydrocoll..

[31] Narmon A.S., Dewaele A., Bruyninckx K., Sels B.F., Van Puyvelde P., Dusselier M. (2021). Boosting PLA melt strength by controlling the chirality of co-monomer incorporation. Chem. Sci..

[32] Wang H., Wang J., Mujumdar A.S., Jin X., Liu Z.-L., Zhang Y., Xiao H.-W. (2021). Effects of postharvest ripening on physicochemical properties, microstructure, cell wall polysaccharides contents (pectin, hemicellulose, cellulose) and nanostructure of kiwifruit (*Actinidia deliciosa*). Food Hydrocoll..

[33] Qin Z., Liu H.-M., Cheng X.-C., Wang X.-D. (2019). Effect of drying pretreatment methods on structure and properties of pectins extracted from Chinese quince fruit. Int. J. Biol. Macromol..

[34] Gawkowska D., Cybulska J., Zdunek A. (2018). Structure-Related Gelling of Pectins and Linking with Other Natural Compounds: A Review. Polymers.

[35] Njoroge D.M., Kinyanjui P.K., Makokha A.O., Christiaens S., Shpigelman A., Sila D.N., Hendrickx M.E. (2014). Extraction and characterization of pectic polysaccharides from easy- and hard-to-cook common beans (*Phaseolus vulgaris*). Food Res. Int..

[36] Gao K., Liu B., Wu B., Guo Y., Song C., Nan S., Dai J., Shen Y., Ma H. (2024). A Study on the Effect Mechanism of Pectin Modification on the Carrot Cell Wall’s Texture Formation under Ultrasonic and Infrared Drying. Agriculture.

[37] Xiao M., Yi J., Bi J., Zhao Y., Peng J., Hou C., Lyu J., Zhou M. (2018). Modification of Cell Wall Polysaccharides during Drying Process Affects Texture Properties of Apple Chips. J. Food Qual..

[38] Feng W., Kita D., Peaucelle A., Cartwright H.N., Doan V., Duan Q., Liu M.-C., Maman J., Steinhorst L., Schmitz-Thom I. (2018). The FERONIA Receptor Kinase Maintains Cell-Wall Integrity during Salt Stress through Ca^2+^ Signaling. Curr. Biol..

[39] Ralet M.-C., Crépeau M.-J., Buchholt H.-C., Thibault J.-F. (2003). Polyelectrolyte behaviour and calcium binding properties of sugar beet pectins differing in their degrees of methylation and acetylation. Biochem. Eng. J..

